# Impact of e-cigarette experimentation and use on smoking behavior among adolescents aged 15–16 years in the Loire department, France

**DOI:** 10.18332/tpc/163416

**Published:** 2023-06-22

**Authors:** André Wamba, Jérémie Pourchez, Julien Masson, Christine Denis-Vatant, Lara Leclerc, Mabrouk Nekaa

**Affiliations:** 1 École normale supérieure de Yaoundé, Département des sciences de l’éducation, Université de Yaoundé I, Yaoundé, Cameroun; 2 École Mines de Saint-Étienne, Université Jean Monnet Saint-Etienne, INSERM, Saint-Etienne, France; 3 Institut national supérieur du professorat et de l’éducation, Université Claude Bernard Lyon 1, INSPÉ, Laboratoire Parcours Santé Systémique P2S, Lyon, France; 4 UCT pôle DocP2 CHU Saint-Étienne, Saint-Étienne, France; 5 Laboratoire ECP, Éducation, Cultures, Politiques, Université Jean Monnet, Saint-Etienne, France

**Keywords:** e-cigarette experimentation and use, smoking experimentation and use, adolescents of 15-16 years, French

## Abstract

**INTRODUCTION:**

We describe the vaping and smoking habits of French adolescents aged 15–16 years in the Loire department with a view to assess the impact of e-cigarette experimentation and use on their smoking behavior.

**METHODS:**

This quantitative, cross-sectional, single-center and observational study conducted from January to July 2019 targeted 6622 students aged 15–16 years attending public high school in the Loire department, France.

**RESULTS:**

A total of 4937 (74.6%) adolescents were included. Of these, 73.2% were non-vapers and 72.2% non-smokers; 66.0% of adolescents were non-vapers and non-smokers. Slightly less than half of adolescents had experimented with e-cigarettes (44.6%), more than half of whom (26.8%) continued to use vaping products, with 6.02% vaping daily. Likewise, a little less than half of adolescents had experimented with smoked tobacco (42.4%), more than half of whom (27.8%) continued to use smoking products, with 10.3% smoking daily. Vapers and smokers (20.6%) tended to begin with the use of smoked tobacco and to progress to the dual use of vaping and smoked tobacco products. Vaping had a positive effect, as 71.8% of vapers who smoked tobacco before initiating vaping stopped or reduced smoking following their progression to this double use. More than half of tobacco users are daily users while this daily use affects only 1/3 boys and 1/6 girls for vape. Finally, nearly 80.7% of adolescents who had never smoked before vaping did not smoke at the time of the study.

**CONCLUSIONS:**

Our data suggest that vaping has a rather marginal impact on smoking initiation among French adolescents aged 15–16 years in the Loire department. They therefore neither confirm nor completely disprove the gateway effect theory, relating to use of tobacco subsequent to vaping.

## INTRODUCTION

Several studies have been conducted on the use of vaping products (e-cigarettes) in France^1-4^. Yet, the specific population of French adolescents has received little attention, except in the studies by Chyderiotis et al.^5,6^ and Stengers et al.^7^. This is unfortunate, as this population is at higher risk of vaping than any other demographic group. Indeed, adolescence is a key stage for the initiation of vaping and smoking in France^8^, as shown by the ‘Baromètre Santé’ 2017 study^1^ and the I-Share study^9^. Many studies examining the progression from smoking to vaping^1,3,10^, and found that there is a strong relationship between tobacco cigarettes and e-cigarettes^7,11-13^. According to some studies^11,14-17^, smokers who attribute a role to e-cigarettes use them as a tool to stabilize, reduce^14^ or stop their use tobacco smoking^18,19^. Yet, the role of vaping in smoking cessation is still debated in the scientific community^16^. Indeed, currently available data appear insufficient to conclude with certainty that e-cigarettes are an effective and safe tool to help smokers wean themselves from smoked tobacco products. Some studies point out that smoking cessation with e-cigarettes may be difficult to achieve in the long-term and that people who succeed in quitting smoking through vaping are not immune to relapse compared to those who rely on nicotine substitutes^14^. Transition from vaping to smoking has been examined through the lens of the gateway theory. According to this theory, vaping is part of a temporal cycle in which the experimentation/use of e-cigarettes is followed by the experimentation and use of smoked tobacco^20^. This theory has given rise to a major scientific controversy, with some studies claiming that non-smokers who use e-cigarettes are more likely to smoke later on and others stating that vaping is not necessarily a gateway to smoking. The controversy is still ongoing, especially since the relationship between e-cigarettes and tobacco products has yet to be thoroughly documented.

The main problem with most available studies is that they analyze the relationship between e-cigarettes and tobacco without properly accounting for the contextual characteristics of smoking and vaping (prevalence, e-cigarette technologies and nicotine content, etc.). Indeed, these characteristics vary greatly from one country to another. For example, the United States has a relatively low smoking prevalence (8.7%)^21^ and a relatively high vaping prevalence among adolescents (11%–21%)^22,23^, the majority of whom prefer using JUUL vaping devices with high nicotine content^24^. By contrast, in France, the smoking prevalence is relatively high (24% in 2020)^25^, with little published data on the prevalence of adolescent vaping or on the most popular vaping technologies. These disparate national environments may explain why some studies suggest the absence of a gateway effect while others appear to prove its existence. Instead of trying to verify the gateway effect theory by assuming its universality, it seems preferable to thoroughly describe the contextual characteristics of vaping and smoking in a given population. A Canadian study has shown, for instance, that the variability of vaping products available on the market^26^ affects the vaping and smoking habits of adolescents. Meanwhile, a study conducted in France^27^ suggests that adolescents may be using e-cigarettes as a way of consuming nicotine or other substances such as cannabis. The fact remains that variations in context make it difficult to determine the vaping or smoking status of adolescents, especially since we do not yet fully understand the trajectories of adolescent dual users, i.e. those who start with vaping and then move on to smoking and those who quit smoking through vaping. In the specific context of France, little knowledge is available on the type of vaping products used by adolescent dual users (e-liquid with or without nicotine) or the role of e-cigarettes among non-smoking adolescents^28^. Our study was conducted in France, where the smoking prevalence remains high compared to other European countries such as the United Kingdom, Germany, and Belgium^29^. We aimed to describe the vaping and smoking habits of adolescents aged 15–16 years attending public high school in the Loire department, with a view to assess the impact of e-cigarette experimentation and use on smoking behavior in this population.

## METHODS

This was an observational, descriptive, cross-sectional and single-center study. It was conducted in partnership with Saint-Etienne University Hospital, the Departmental Services of National Education of the Loire, and the École Nationale Supérieure des Mines de Saint-Etienne.

The study targeted the population of adolescents aged 15–16 years attending one of the 27 public high schools of the Loire department in 2019 (n=6622). To recruit participants, an informational note was sent by the administration of each high school to parents and students before the questionnaire was administered. This note stated that participation in the survey was voluntary and that in order to complete the questionnaire, students had to be present in a computer classroom reserved by the high school administration. Data collection was carried out using a self-administered questionnaire developed by a multidisciplinary team. During the design phase of the questionnaire, preliminary reliability and validity tests were conducted with some adolescents who were not included in the study sample, to ensure proper understanding of the questions. Designed and created online using Google Forms, the questionnaire was mainly composed of closed-ended questions (Supplementary file), with some semi-open-ended questions on the vaping and smoking habits of the social circle, the e-liquid flavors used etc., and with one ‘other’ question where respondents could add any comments they wished. Data collection met the anonymity requirements established by the National Commission for Information Technology and Liberties (CNIL), and were split into four main categories:

1. Basic characteristics of study participants: gender, age and school level, characteristics of members of the social circle (parents # 1 and # 2, siblings and school friends);
2. Experimentation and use of smoked tobacco, with a focus on the type of tobacco products used, smoking duration, smoking frequency and smoking status of members of the social circle;
3. Experimentation and use of e-cigarettes, with a focus on the type of vaping products used, vaping duration, vaping frequency and vaping status of members of the social circle; and
4. Impact of e-cigarette experimentation and use on smoking behavior: temporal prevalence of vaping and smoking, progression from vaping to smoking, variation in tobacco use in dual users who began with smoking and then moved on to vaping.

The questionnaire was proposed to the adolescents by the school nurse and administrated in computer classrooms (in the presence of a teacher or the nurse but without active supervision) of each high school included in the studies with the support of the National Education of the Loire Department. The questionnaire was administered from January to July 2019, at a date and time decided in consultation with the school principal. The objectives of the study were clearly presented to the students as well as to the nurses and teachers who were to help them fill in the questionnaire in the computer classrooms. During questionnaire completion, the students were seated alone at a desk to guarantee anonymity, to facilitate reading and to ensure that answers were personal. Each student filled in the questionnaire online via the URL link provided on a digital tablet, with a maximum time of 3–15 minutes.

Smoker status was assigned to adolescents who reported smoking tobacco or cannabis daily or occasionally. Non-smokers were adolescents who had never experimented with smoked tobacco before the time of the study. Adolescents who had smoked before the start of the study and those who had experimented with smoked tobacco only once were classified as ex-smokers. Similarly, vaper status was assigned to adolescents who reported vaping daily or occasionally. Non-vapers were adolescents who had not experimented with e-cigarettes before the start of the study. Adolescents who had vaped before the time of the study and those who had experimented with e-cigarettes only once were classified as exvapers.

### Statistical analysis

Questionnaires were processed using Excel® software. Statistical analyses were performed using IBM SPSS Statistics 21® software. Percentages were compared in univariate analysis using the chi-squared test. Averages were compared using an analysis of variance or the non-parametric Kruskal-Wallis test, as appropriate. Pearson’s correlation coefficient was used to measure the association between quantitative variables.

## RESULTS

Of the 6622 students aged 15–16 years, 4937 students participated in the study (50.5% girls and 49.5% boys), which corresponds to nearly three-quarters (74.6%) of the target population.

### Smoking: experimentation and use of smoked tobacco

**Figure 1:**
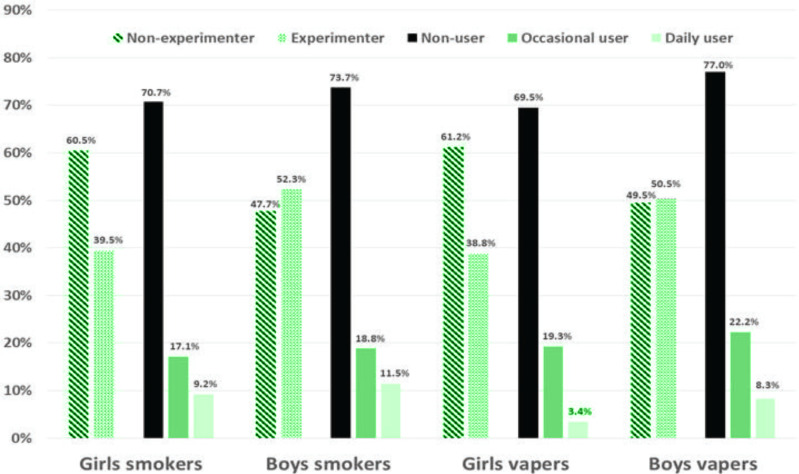
Smoking and vaping prevalence according to sex

Overall, the results show that 42.4% of adolescents had experimented with smoked tobacco, with more boys (52.3%) reporting this behavior than girls (39.5%) (p<0.001) (Figure 1 and Table 1). Of these, more than half continued to smoke daily or occasionally. Thus, 27.8% of adolescents reported being smokers (the questions used to define the smoking status of adolescents are presented in Table 2), with more boys (29.3%) doing so than girls (26.3%) (p=0.02) (Figure 1 and Table 1). The prevalence of occasional smoking was 17.4% and was almost equal between boys and girls (17.8% and 17.1%, respectively). The prevalence of daily smoking was 10.3%, with more boys (11.5%) reporting this behavior than girls (9.2%). This means that nearly one-third of tobacco users were daily smokers. The most commonly used tobacco products were tobacco cigarettes (14.4%) and hookah (13.7%), followed by rolled cigarettes (9.6%), smoked cannabis (7.6%) and cigars/cigarillos (2.9%).

**Table 1:** Differences between the rate of occasional use and daily use

*Use status*	*Smokers %*		*Vapers %*	
	*Girls*	*Boys*	*Girls*	*Boys*
Non-experimenter	60.5	47.7	61.2	49.5
Experimenter	39.5	52.3	38.8	50.5
Non-user	70.7	73.7	69.5	77.0
Occasional user	17.1	18.8	19.3	22.2
Daily user	9.2	11. 5	3.4	8.3
Rate of daily users among users	54.1	60.8	17.9	37.3

**Table 2:** Smoking status of adolescents and smoking prevalence in the study population

*Use of smoking products*	*Smoking status*		*Experimentation*	
	*Smoker*	*Non-smoker*	*Yes*	*No*
Uses one of the following smoking products: tobacco cigarettes, hand-rolled cigarettes, cigars/cigarillos, hookah	√		√	
Reduced his/her use of smoking products, even if he/she reports smoking only on weekends or exceptionally	√			
Smokes only cannabis	√		√	
Stopped using smoking products		√	√	
Never smoked tobacco or cannabis products		√		√
Smoking prevalence in the study population	27.8% (including 17.4% occasional use and 10.3% daily use)	72.2%	42.4%	57.6 %

More than half of the adolescents (56.6%) had first experimented with smoked tobacco at least one year before the study. Likewise, more than half (53.1%) had experimented with hookah smoking at least one year before the study. These findings indicate that adolescents’ initiation to the most popular smoked tobacco products (cigarettes and hookah) takes place mainly at the age of 14–15 years. Finally, smokers were significantly more likely to be boys than girls (p=0.02) (Figure 1 and Table 1).

### Vaping: experimentation and use of e-cigarettes

**Figure 2:**
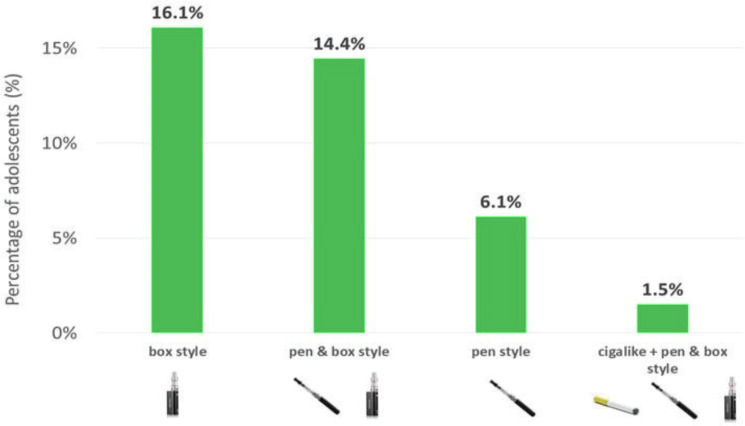
Frequency of use of vaping products among adolescents

The questions used to define the vaping status of adolescents are presented in Table 3. Overall, the results show that 44.6% of adolescents had experimented with e-cigarettes, with more boys (50.5%) reporting this behavior than girls (38.8%) (p<0.001) (Figure 1 and Table 1). Of these, more than half continued to vape daily or occasionally. Thus, 26.7% of adolescents reported using e-cigarettes, with more boys (30.5%) doing so than girls (23.0%). The prevalence of occasional vaping was 20.7% and was slightly higher in boys (22.2%) than girls (19.3%). The prevalence of daily vaping was 6.0%, with more boys (8.3%) reporting this behavior than girls (3.8%). This means that less than one-quarter of e-cigarette users were daily vapers (p<0.001) (Figure 1 and Table 1). The most commonly used vaping products were, in order: box style (16.1%); pen style and box style (14.4%); pen style (6.1%); and cigar like style, pen style and box style (1.5%) (Figure 2 and Table 1). The study was conducted before the puff-bar epidemic that started in France in 2021.

**Table 3:** Vaping status of adolescents and vaping prevalence in the study population

*Use of vaping products*	*Vaping status*		*Experimentation*	
	*Vaper*	*Non-vaper*	*Yes*	*No*
Uses one of the following vaping products: box style, pen style, POD style, JUUL style	√		√	
Reduced his/her use of vaping products, even if he/she reports vaping only on weekends or exceptionally	√		√	
Uses only box style	√			
Stopped using vaping products		√	√	
Never used vaping products		√		√
Vaping prevalence in the study population	26.8% (including 20.7% occasional use and 6.0% daily use)	73.2%	44.6%	55.4 %

Of the adolescents who reported vaping, the majority (54.9%) had first experimented with vaping products in the year prior to the study. Half (45.4%) of the adolescents who had experimented with e-cigarettes had never experimented with smoked tobacco before, suggesting that e-cigarettes are one of the main initiation products. Most of the adolescents (77.9%) who had experimented with e-cigarettes stated that the device was not theirs (‘it wasn’t mine’). Of those who owned the device (22.1%), more than half had bought it in a physical store (56.2%), about a third had received it as a gift (31.4%), and a minority had purchased it on the Internet (12.2%). Finally, the statistical analysis of the characteristics of experimenters and users of e-cigarettes showed that vapers were significantly more likely to be boys than girls (p<0.001) (Figure 1 and Table 1).

### Relationship between vaping and smoking among adolescents

**Figure 3:**
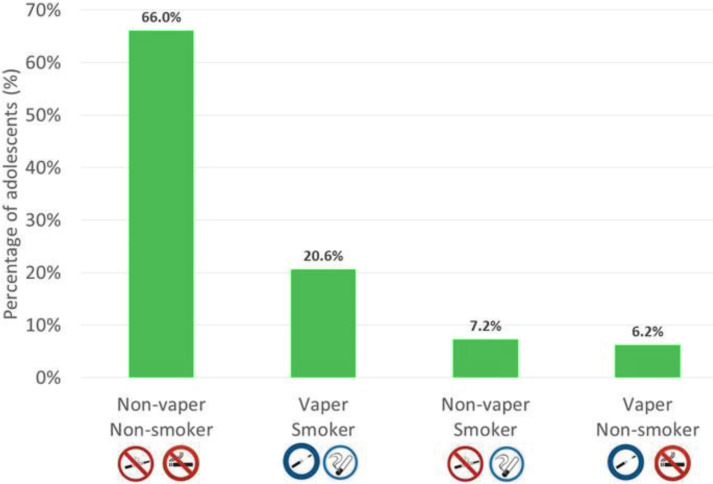
Smoking and vaping status of adolescents

Adolescents were classified into four categories according to their smoking and vaping habits: ‘non-vaper and non-smoker’, ‘vaper and smoker’, ‘non-vaper and smoker’, and ‘vaper and non-smoker’. The majority of vapers were also smokers and vice versa. Thus, 74.0% of smokers were also vapers (20.6% of ‘smokers and vapers’ vs 7.2% of ‘smokers and non-vapers’) and 76.8% of vapers were also smokers (20.6% of ‘vapers and smokers’ vs 6.2% of ‘vapers and non-smokers’) (Figure 3).

Our analysis of the temporal prevalence of vaping and smoking among adolescents aged 15–16 years found that ‘vapers and smokers’ (20.6%) tended to begin with the use of smoked tobacco and to progress to the dual use of vaping and smoked tobacco products. This is perfectly consistent with the reported period of initiation of smoking and vaping, i.e. experimentation with smoked tobacco mostly at 14 years and with e-cigarettes mostly at 15 years. Thus, our data suggest that there is no major gateway effect until the age of 15–16 years. It also appears that the fraction of adolescents aged 15–16 years who may be concerned by the gateway effect in the near future is extremely low, as only 6.2% reported being ‘vapers and non-smokers’ (Figure 3).

Nearly half (45.6%) of the adolescents who had initiated vaping did not know the nicotine content of their e-cigarettes or whether these contained nicotine. Moreover, 21.8% declared using mostly nicotine-free e-cigarettes (31.4% of those who had never smoked tobacco before initiating vaping vs 13.6% of those who had smoked tobacco before initiating vaping) and 32.7% reported using e-cigarettes with a nicotine content ranging 0–12 mg/mL (20.1% of those who had never smoked tobacco before initiating vaping vs 42.9% of those who had smoked tobacco before initiating vaping). These data suggest that the nicotine content of e-cigarettes strongly depends on whether adolescents had smoked before initiating vaping.

### Impact of the social circle on initiation of smoking and vaping among adolescents

The smoking and vaping status of the social circle appears to have a strong impact on the smoking and vaping status of adolescents aged 15–16 years. Indeed, when no member of their social circle smoked, the percentage of adolescents who smoked daily or occasionally was extremely low (2.2%) (Table 4). In contrast:

**Table 4:** Impact of the social circle on the smoking status of adolescents (only responses with a frequency of greater than 1.5% were considered) (N=4490)

*Smoking prevalence in adolescents’ social circle*	*Smoking status of adolescents*		*Frequency of response n*
	*Smoker %*	*Non-smoker %*	
Nobody smokes	2.2	97.8	1155
Both parents smoke	3.9	96.1	102
Other household members smoke	5.9	94.1	170
One parent smokes	6.2	93.8	433
Siblings smoke	8.5	91.5	118
Other household members + friends smoke	13.5	86.5	192
Friends smoke	13.6	86.4	774
One parent + friends smoke	17.2	82.8	360
One parent + other household members + friends smoke	18.5	81.5	108
Both parents + friends smoke	20.2	79.8	89
One parent + siblings + friends smoke	25.0	75.0	72
Siblings + friends smoke	26.9	73.1	173
Both parents + other household members + friends smoke	37.8	62.2	90
Both parents + siblings + other household members + friends smoke	61.7	38.3	94

- Between 25.0% and 61.7% of adolescents smoked when members from at least three categories of their social circle (parents, friends, siblings, etc.) smoked;
- Between 17.2% and 20.2% of adolescents smoked when members from two categories of their social circle (parents, friends, siblings, etc.) smoked; and
- Between 3.9% and 13.6% of adolescents smoked when members from only one category of their social circle smoked.

Likewise, only 8.7% of adolescents vaped when no member of their social circle vaped (Table 5). In contrast:

- Between 54.4% and 63.8% of adolescents vaped when members from at least three categories of their social circle (parents, friends, siblings, etc.) vaped;
- Between 38.9% and 54.3% of adolescents vaped when members from two categories of their social circle (parents, friends, siblings, etc.) vaped; and
- Between 14.8% and 33.5% of adolescents vaped when members from only one category of their social circle vaped.

## DISCUSSION

Our objective was not to conduct a study that would be representative of the smoking and vaping behavior of French adolescents. Indeed, we aimed at carrying out a study representative of the smoking and vaping behavior of adolescents from the Loire department at the age of 15–16 years. Thus, we included approximately 75% of this population in the end. Our initial ambition was to have a maximum rate to be representative of this population, the teenagers of the Loire department in France. Thus, we have a good representativeness of our target population. Nevertheless, even if our objective is not to be representative of French adolescents, the Loire department has characteristics that are quite similar (especially sociodemographic) to the whole of France, so it is not very surprising that the prevalence results we obtained are consistent with the French national prevalence.

### Prevalence of smoking and vaping among adolescents

In our study, 27.8% of the surveyed adolescents were smokers. Recent studies on French adolescents of similar age reported comparable data: the 2015 ESPAD report^30^ found a prevalence of 26% in French students aged 15 years^6^, while the 2018 study by Denis-Vatant et al.^12^ reported a prevalence of 28.2% in a population of French adolescents aged 15–16 years. Our study also found smoking prevalence to be higher in boys (45.3%) than girls (39.5%), a trend observed in other French studies^27^.

The prevalence of vaping in our study population was 44.6%. As in the case of smoking, boys (50.5%) were more likely than girls (38.8%) to experiment with e-cigarettes. The latter finding is in line with studies evaluating the vaping behavior of adolescents of similar age as in our study^5,15,31,32^. For instance, in the study by Chyderiotis et al.^5^ on French adolescents aged 17 years, the prevalence of e-cigarette experimentation was high in both boys (56.5%) and girls (48.1%). Interestingly, similar prevalence figures were reported in the French adult population^33^. Furthermore, in our study, the prevalence of e-cigarette experimentation (44.7%) was close to that of tobacco experimentation (42.4%). These figures, however, are slightly lower than those reported in the 2017 ESCAPAD study^27^ for e-cigarettes (52.4%) and tobacco (59.0%) experimentation, which may be explained by the fact that the students surveyed in the ESCAPAD study were slightly older (17 years) than in our study (15–16 years). By contrast, the percentage of daily vapers in our study (6.0%) was about three times higher than that reported (1.9%) in the ESCAPAD study^27^. Moreover, it was about twice as high as that reported in the studies by Stenger et al.^7^ for middle and high school students (3.4%) and that by Denis-Vatant et al.^12^ for adolescents of the same age as in our study (15–16 years) (3.7%). That said, few of the adolescents who had experimented with e-cigarettes in our study progressed to daily vaping. This may be explained by the fact that adolescents who initiate vaping are simply looking for new experiences, as some studies indicate^12,34^. Nevertheless, the percentage of daily vapers in our study is consistent with international studies that show a rapid increase in e-cigarette use in the adolescent population – with the latest US data reporting a twofold increase in school-based e-cigarette use between 2017 and 2018^35^.

### Relationship between e-cigarette use and smoked tobacco use

Our study found that nearly three in four adolescent smokers (74.0%) were also e-cigarette users (while 27.8% of adolescents were ‘smokers’, 20.6% were ‘smokers and vapers’ and 7.2% were ‘smokers and non-vapers’) (Figure 3). Likewise, more than three in four adolescent vapers (76.8%) were also users of smoked tobacco products (while 26.8% of adolescents were vapers, 20.6% were ‘vapers and smokers’ and 6.2% were ‘vapers and non-smokers’) (Figure 3). In other words, most smokers and/or vapers engaged in dual use of e-cigarettes and smoked tobacco products. In the study by Chyderiotis et al.^5^, e-cigarette use among French adolescents was also generally associated with daily use of smoked tobacco (62.5%), even if it remained limited compared to e-cigarette experimentation. In fact, French1^3,27^ and international^35^ studies suggest that vaping may play a role in delaying or preventing smoking among nonsmokers, in stopping or reducing tobacco consumption in dual users, and even in protecting former smokers from relapse by allowing them to develop exclusive vaping behavior. It therefore seems important to trace the smoking and vaping history of single and dual users, and to identify the factors associated with vaping and smoking among adolescents in France.

### Temporal prevalence of vaping and smoking: gateway effect and impact of vaping on smoked tobacco use

First, we sought to determine the magnitude of the gateway effect in our study population. We found that only 535 adolescents were vapers who had never smoked tobacco before initiating vaping. Of these, 19.3% reported being smokers (n=103) and 80.8% reported being non-smokers (n=432; 300 had never smoked and 132 were ex-smokers). Thus, 103 adolescents began with the use of e-cigarettes and subsequently progressed to the dual use of vaping and smoked tobacco products, giving a percentage of 2.2% of surveyed adolescents influenced by the gateway effect at the time of the study. Moreover, 132 adolescents began with the use of e-cigarettes, subsequently progressed to the dual use of vaping and smoked tobacco products and eventually quit smoking, meaning that 2.7% of surveyed adolescents were influenced by the gateway effect prior to the study. In short, the global impact of the gateway effect in our study population was relatively modest, as only 4.8% of French adolescents aged 15–16 years progressed from vaping to smoking (with 2.2% influenced by the gateway effect at the time of the study and 2.7% prior to the study). This finding, along with the facts that vaping was rare among non-smoking adolescents (6.2%) and that the prevalence of tobacco use (42.4%) was lower than that of e-cigarette use (44.6%), supports other studies which found no major gateway effect in the French population^5,13,27^.

Second, we sought to assess the impact of vaping on tobacco use in our study population. We found that 1107 adolescents were vapers who had smoked tobacco before initiating vaping. Vaping had a positive effect in 71.8% of these adolescents (n=795), as 47.9% quit smoking (n=530) and 23.9% reduced their tobacco use after initiating vaping (n=265). By contrast, 19.2% of these adolescents maintained their tobacco use after initiating vaping (n=213), indicating no effect of vaping on their smoking behavior. Finally, only 8.9% of these adolescents increased their tobacco use after initiating vaping (n=99), suggesting that vaping had a negative effect on their smoking behavior.

### Limitations

Our study has several limitations. The use of cross-sectional and descriptive data made it impossible to perform a prospective analysis. However, our research team is currently conducting longitudinal cohort studies in France to explore the temporal evolution of vaping and smoking behavior (including smoking cessation) between the ages of 15 and 18 years and to determine the relationship between e-cigarette use and tobacco use during adolescence. Furthermore, since our sample was limited to adolescents aged 15–16 years in the Loire department, our findings cannot be directly extrapolated to other populations.

## CONCLUSIONS

The above findings suggest that e-cigarette use does not strongly favor smoking in French adolescents aged 15–16 years. However, it cannot be excluded that a considerable number of adolescents who reported being ‘vapers and non-smokers’ will initiate smoking in the coming years. A major finding of our study is that vaping had a positive effect on the smoking behavior of French adolescents aged 15–16 years, with many quitting or reducing their tobacco use after initiating vaping. In our study, the low percentage of adolescents who reported using e-cigarettes before initiating smoking and the significant percentage of non-smokers who used nicotine-free e-cigarettes do not support the existence of a gateway effect. This is in line with many studies conducted in the school setting in which vaping was not a gateway to smoking^5,7,13,20,35-37^.

## Data Availability

The data supporting this research are available from the authors on reasonable request.
