# Supplementary material for: Impact of e-cigarette experimentation and use on smoking behavior among adolescents aged 15–16 years in the Loire department, France
Source: Tob Prev Cessat. 2023 Jun 22;9:21. doi: 10.18332/tpc/163416 (PMC10286514; doi:10.18332/tpc/163416)
Supplement: Supplementary file 1 [file TPC-9-21-s1.pdf]

SUPPLEMENRARY DATA

questions		Possible answers
General questions (posed to all)	**Gender**	Boy / Girl
	**Name of school**	choice among the +20 schools participating in the study
	**Class level**	Years 11 pupils
	**Status**	Internal / External (never eats at the canteen) / Half boarder (eat canteen occasionally or regularly)
	**Profession of "parent 1"**	Farmers / Craftsmen, traders and entrepreneurs / Managers / Intermediate Professions / Workers / Pensioners / Without profession / I don't know about it or not concerned
	**Profession of "parent 1"**	Farmers / Craftsmen, traders and entrepreneurs / Managers / Intermediate Professions / Workers / Pensioners / Without profession / I don't know about it or not concerned
	**Monthly money available (pocket money or salary)**	None / Between 1 and 20 € / Between 21 and 50 € / Between 51 and 100 € / More than 100 €
Smoking questions (posed to all)	**In your close circle, who smokes? (Several possible answers)**	No one / Parent 1 / Parent 2 / Your Brother or sister / The entourage you live / your friends / other: free field to be completed
	**You have already smoked one of the following products: cigarette, rolled cigarette, cigar or cigarillo, chicha or cannabis**	Yes / No
Questions asked only to users and experimenters of smoked tobacco products	**Have you ever smoked cigarettes?**	No / only once / several times / Regularly (at least once a week)
	**Have you ever smoked rolled cigarettes?**	No / only once / several times / Regularly (at least once a week)
	**Have you ever smoked cigars or cigarillos?**	No / only once / several times / Regularly (at least once a week)
	**Have you ever smoked chicha?**	No / only once / several times / Regularly (at least once a week)
	**Have you ever smoked cannabis?**	No / only once / several times / Regularly (at least once a week)
	**What are you doing now for cigarettes?**	You've never smoked any / You stopped / You've reduced / You continue
	**What are you doing now for rolled cigarettes?**	You've never smoked any / You stopped / You've reduced / You continue
	**What are you doing now for cigars and cigarillos?**	You've never smoked any / You stopped / You've reduced / You continue
	**What are you doing now for chicha?**	You've never smoked any / You stopped / You've reduced / You continue
	**What are you doing now for cannabis?**	You've never smoked any / You stopped / You've reduced / You continue
	**When is your first try/1st use of cigarettes?**	Never / < 1 month / 1 to 6 months / 6 months to 1 year / 1 to 2 years / > 2 years
	**When was your first try/1st use of rolled cigarettes?**	Never / < 1 month / 1 to 6 months / 6 months to 1 year / 1 to 2 years / > 2 years
	**When was your first try/1st use of cigarettes and cigarillos?**	Never / < 1 month / 1 to 6 months / 6 months to 1 year / 1 to 2 years / > 2 years
	**When was your first try/1st use of chicha?**	Never / < 1 month / 1 to 6 months / 6 months to 1 year / 1 to 2 years / > 2 years

	**When was your first trial/1st use of cannabis?**	Never / < 1 month / 1 to 6 months / 6 months to 1 year / 1 to 2 years / > 2 years
	**Why did you smoke? (Several possible answers)**	Out of curiosity / To appear more adult / To make smoke / To look cooler / To do like the others / Other: free field to be completed
	**What do you like about tobacco? (Several possible answers)**	The taste / Smoking with your friends / The passage of smoke in the throat / Making Smoke / It relaxes or destresses / I didn't like anything / Other: free field
	**How did you get your tobacco products? (Several possible answers)**	Tobacco office / Family / Friends / Other: free field to be completed
	**How often do you smoke?**	Every day / Only the week / Only on weekends / Exceptionally (in the evening or travel for example) / I don 't smoke or I don't smoke anymore
**about vaping (posed to**	**In your close circle, who's vaping? (Several possible answers)**	No one / Parent 1 / Parent 2 / Your Brother or sister / The entourage you live / your friends / Other: free field to be completed
	**Have you ever vaped?**	No / only once / several times / Regularly (at least once a week)
**Questions asked only to users and experimenters of vaping products**	**When does your first try/1st use of e-cigarette?**	< 1 month / 1 to 6 months / 6 months to 1 year / 1 to 2 years / > 2 years
	**Which e-cigarette have you ever used? (Several possible answers with photo to illustrate the device)**	cigalike type / Pen style type / box type / JUUL POD type / heated tobacco devices e.g., iqos or PLOOM / Other: free field to be completed
	**Regarding the e-cigarette you used (Several possible answers)**	It's yours / It belongs to a friend / It belongs to a member of your family / Other: free field to be completed
	**How did you get that e-cigarette? (Several possible answers)**	"Gift" from a friend / Tobacco office / Vape Shop / Internet / "Gift" from a member of your family / It's not mine
	**How often are you vaping?**	Every day / Only the week / Only on weekends / Occasionally (e.g., evening or travel) / I don't vape or I don't vape anymore
	**For what reason(s) did you vape? (Several possible answers)**	Out of curiosity / To appear more adult / To make smoke / To look cooler / To do like the others / To reduce or stop my tobacco use / To save money compared to the cost of tobacco / for the diversity of flavors/Other: free field to be completed
	**Why are you still vaping? (Several possible answers)**	To reduce or stop my tobacco use / To do like the others / To appear more adult / To save money compared to tobacco costs / To look cooler / To make smoke / For the diversity of flavors / I'm not vaping or not vaping anymore / Other: free field to be completed
	**Before vaping for the first time, had you ever smoked cigarettes?**	Yes / No / I tried both at the same time
	**What is your cigarette consumption today?**	I quit smoking cigarettes / My cigarette consumption is down / My cigarette consumption is stable / My cigarette consumption is on the rise / I've never smoked
	**What are the nicotine dosages of e-liquid vials that you have already used? (Several possible answers)**	I don't know - Zero mg/mL- 3 mg/mL - 6 mg/mL - 12 mg/mL - 16 mg/mL or more
	**What are the aromas you've ever used? (Several possible answers)**	Fruit or other sweet tastes / Taste tobacco / Mint / Other: free field to be completed
	**What did you like about the e-cigarette? (Several possible answers)**	The taste /Vaping with your friends / The passage of smoke in the throat / Making Smoke / It relaxes or destresses / The technological aspect / I didn't like anything /Other: free field to be completed

Supplementary data: Translation (from French to English) of the questionnaire used for the study

© 2023 Wamba A. et al.
